# Combining Turing and 3D vertex models reproduces autonomous multicellular morphogenesis with undulation, tubulation, and branching

**DOI:** 10.1038/s41598-018-20678-6

**Published:** 2018-02-05

**Authors:** Satoru Okuda, Takashi Miura, Yasuhiro Inoue, Taiji Adachi, Mototsugu Eiraku

**Affiliations:** 1 RIKEN Center for Developmental Biology, 2-2-3 Minatojima-minamimachi, Chuo-ku, Kobe, Hyogo 650-0047 Japan; 2 PRESTO, Japan Science and Technology Agency, 4-1-8 Honcho, Kawaguchi, Saitama 332-0012 Japan; 3 Faculty of Medical Sciences, Kyushu University, 3-1-1 Maidashi, Higashi-ku, Fukuoka, 812-8582 Japan; 4 Institute for Frontier Life and Medical Sciences, Kyoto University, 53 Kawahara-cho, Shogoin, Sakyo-ku, Kyoto, 606-8507 Japan

## Abstract

This study demonstrates computational simulations of multicellular deformation coupled with chemical patterning in the three-dimensional (3D) space. To address these aspects, we proposes a novel mathematical model, where a reaction–diffusion system is discretely expressed at a single cell level and combined with a 3D vertex model. To investigate complex phenomena emerging from the coupling of patterning and deformation, as an example, we employed an activator–inhibitor system and converted the activator concentration of individual cells into their growth rate. Despite the simplicity of the model, by growing a monolayer cell vesicle, the coupling system provided rich morphological dynamics such as undulation, tubulation, and branching. Interestingly, the morphological variety depends on the difference in time scales between patterning and deformation, and can be partially understood by the intrinsic hysteresis in the activator-inhibitor system with domain growth. Importantly, the model can be applied to 3D multicellular dynamics that couple the reaction–diffusion patterning with various cell behaviors, such as deformation, rearrangement, division, apoptosis, differentiation, and proliferation. Thus, the results demonstrate the significant advantage of the proposed model as well as the biophysical importance of exploring spatiotemporal dynamics of the coupling phenomena of patterning and deformation in 3D space.

## Introduction

During morphogenesis, cells express various mechanical behaviors according to their chemical states, such as protein synthesis, mRNA transcription, and gene methylation. The local cell states are regulated by global tissue patterning, which is caused by chemical interactions among multiple cells; for example, signaling molecules diffuse away from local source cells and provide a steady gradient within a tissue^1–3^. Moreover, adding chemical reactions to molecular diffusions can generate various complex patterns due to the Turing instability^4–6^. Importantly, because signaling molecules are transported inside 3D-structured tissues, chemical patterning occurs in their 3D geometry - i.e., the single cell shape, multicellular configuration, and entire tissue shape. By focusing on the 3D geometry, recent studies have reported patterning processes^7,8^, and those coupled with deformations in 3D space^9,10^.

Based on chemical patterning, cell behaviors can be regulated at a single cell level; for example, Notch–Delta interactions can express different chemical states between neighboring cells^11^. Depending on their chemical states, individual cells express various cell activities such as contraction, adhesion, migration, proliferation, and apoptosis^12^. For example, in the developmental process of mouse palatal shelve, the fibroblast growth factor (FGF) and Sonic hedgehog (Shh) compose an activator-inhibitor system, and operate growth regions in the 3D structure of embryo^13^. These cell activities are coordinated to drive global tissue deformations, and cause local changes in the cell mechanical state, such as cell shape, size, and stress. Simultaneously, the local changes in the cell mechanical state can trigger further molecular signaling^14^. Local cell dynamics can therefore be coupled with global tissue dynamics, forming a basis of bidirectional interaction between patterning and deformation at a single cell level.

Mathematical models have been well used for understanding multicellular dynamics^15–22^ and have been improved to analyze their 3D dynamics^23–28^. We have developed a full 3D vertex model that expresses 3D multicellular dynamics compacted in a monolayer sheet as well as a multilayer aggregate, involving cell rearrangements^29^, division^30^, apoptosis^31^, and viscoelastic behaviors^32^. The models have succeeded in reproducing basic epithelial deformations^33,34^ as well as reproducing several developmental phenomena, such as blastocyst formation^35^. Notably, although the intercellular transport of signaling molecules has been expressed in a 3D vertex model^36^, it has not yet been applied to complex patterning caused by reaction–diffusion dynamics. Therefore, combining the Turing and 3D vertex models will aid in the exploration of mechanochemical coupling in multicellular morphogenesis.

In this study, we propose a novel mathematical model that combines the Turing and 3D vertex models, and demonstrate computational simulations of complex phenomena emerging from the coupling of patterning and deformation, in 3D space. In embryogenesis, diffusive molecules can be transduced to various cell behaviors such as deformation, rearrangement, division, apoptosis, differentiation, and proliferation. As an example, an activator–inhibitor system is assumed as a regulatory process of cell proliferation, and local activator concentration is converted into the growth rate of individual cells. By examining the physical parameters of molecular transport coefficients, production and degradation rates, and cell growth rate, we discuss bidirectional effects occurring between patterning and deformation.

## Model Framework of Combining Turing and 3D Vertex Models

**Figure 1:**
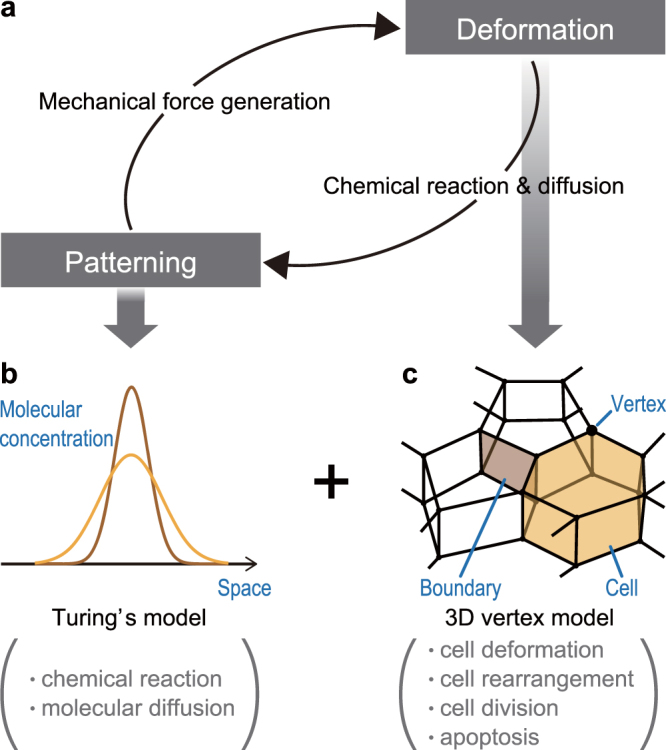
Model framework of combining the Turing and 3D vertex models. (**a**) Concept model framework for coupling multicellular patterning and deformation. (**b**) Turing model for expressing spatial patterning. (**c**) Vertex model for expressing multicellular deformation.

To analyze 3D multicellular dynamics coupling chemical patterning with mechanical deformation, we develop a mathematical model that combines the Turing and 3D vertex models (Fig. 1a). The Turing model is well known to generate various chemical patterns observed in biological phenomena (Fig. 1b), while the 3D vertex model is a general tool to express mechanical behaviors of 3D multicellular dynamics (Fig. 1c). In the combined model, chemical states of individual cells are regulated by chemical interactions among cells; individual cells generate mechanical forces to deform the tissue according to these chemical states. Simultaneously, the chemical pattern can be dynamically rearranged on the deforming tissue, so as to rewrite the chemical states of individual cells. The combined model therefore enables expression of mechanochemical coupling of patterning and deformation. Details of the combined model framework are described below.

### 3D vertex model for describing multicellular deformation

In the 3D vertex model, an individual cell shape is represented by a polyhedron, a boundary face between neighboring cells expressed by a polygon, and the entire structure of a 3D cell aggregate expressed by a single network (Fig. 1c). Topology of the network is dynamically rearranged to express the changes in the cell configuration and the number of cells^29^.

According to the force balance, the movement of the *i*th vertex, represented by ***r***_*i*_, is expressed by the following equation:

1 $${\eta }_{i}(\frac{{\rm{d}}{{\boldsymbol{r}}}_{i}}{{\rm{d}}t}-{{\boldsymbol{V}}}_{{\rm{f}}i})=-{\nabla }_{i}U$$

The left hand side of Eq. (1) indicates a viscous friction force exerted on the *i*th vertex, where scalar *η*_*i*_ and ***V***_f*i*_ are a viscous friction coefficient of the *i*th vertex and vector and a local velocity field around the *i*th vertex, respectively. Here, *η*_*i*_ and ***V***_f*i*_ are defined as functions of velocities of vertices to express the viscous property^32^. The right hand side of Eq. (1) indicates an energetic force acting on the *i*th vertex, where *U* is an effective energy. By supposing *U* as a function of ***r***_i_ and molecular concentration, the mechanical deformation of cells depends on their chemical patterning.

### Discrete Turing model for describing multicellular patterning

Although the Turing model is often described in a continuum manner, in biological systems, molecules diffuse within a multicellular structure. For simplification, as an example, we assume that (i) the molecules transport within cells through junctional structures but not the outside of cells, (ii) the molecular diffusivity within individual cells is much faster than those between neighboring cells, and thereby the molecular concentrations within individual cells can be regarded to be homogeneous, (iii) the gap width between neighboring cells is homogeneous and the molecular distribution between neighboring cells is linear in the normal direction to the cell–cell boundary. Based on these assumptions, the molecular distribution is discretized by individual cell compartments, and the molecular flux between neighboring cells can be simply expressed to be proportional to the difference in the molecular concentrations. This discrete description of the Turing model in the single cell level can be directly combined with the 3D vertex model.

According to the mass conservation, the dynamics of the number of the ${\mathcal M}$th molecules at the *j*th cell, represented by ${m}_{ {\mathcal M} j}$, is expressed by the following equation:

2 $$\frac{{\rm{d}}{m}_{ {\mathcal M} j}}{{\rm{d}}t}={\mu }_{ {\mathcal M} }\sum _{k(j)}^{{\rm{cell}}}(\frac{{m}_{ {\mathcal M} k}}{{v}_{k}}-\frac{{m}_{ {\mathcal M} j}}{{v}_{j}}){s}_{jk}^{{\rm{cc}}}+{v}_{j}{R}_{ {\mathcal M} }({\{\frac{{m}_{ {\mathcal M} j}}{{v}_{j}}\}}_{ {\mathcal M} })\mathrm{.}$$

The first term of the right hand side of Eq. (2) is the flux from the cells neighboring the *j*th cell, where ${\sum }_{k(j)}^{{\rm{cell}}}$ is the summation for all neighboring cells. Constant ${\mu }_{ {\mathcal M} }$ is the transport rate of the ${\mathcal M}$th molecules between neighboring cells as the diffusion coefficients divided by the gap width. Scalar ${s}_{jk}^{{\rm{cc}}}$ is the boundary area between the *j*th and *k*th cells, and scalar *v*_*j*_ is the *j*th cell volume. Both ${s}_{jk}^{{\rm{cc}}}$ and *v*_*j*_ are functions of the location vector of vertices. The second term of the right hand side of Eq. (2) is the reaction term at the *j*th cell, where function ${R}_{ {\mathcal M} }$ is a reaction rate of the ${\mathcal M} {\rm{th}}$ molecules as a function of a set of molecular concentrations at the *j*th cell, represented by ${\{{m}_{ {\mathcal M} j}/{v}_{j}\}}_{ {\mathcal M} }$. Because the flux in the first term depends on the network topology and vertex locat_i_ons ***r***_i_, the chemical patterning of cells depends on their mechanical deformation.

Notably, while Eq. (2) is similar to those in our previous study^36^, the second term in Eq. (2) is newly defined as a function of a set of molecular concentrations in a discrete manner to combine the Turing and 3D vertex models. The relationship between discrete and continuum Turing models is described in Sect. 6 in Appendix.

In this model, the mechanical deformation and the chemical patterning of multiple cells are thereby coupled bidirectionally. The total degrees of freedom of a tissue are represented by the network topology, vertex locations, ***r***_*i*_, and the number of molecules, ${m}_{ {\mathcal M} j}$.

## Modeling Example: Cell Growth Regulation by an Activator–inhibitor System

**Figure 2:**
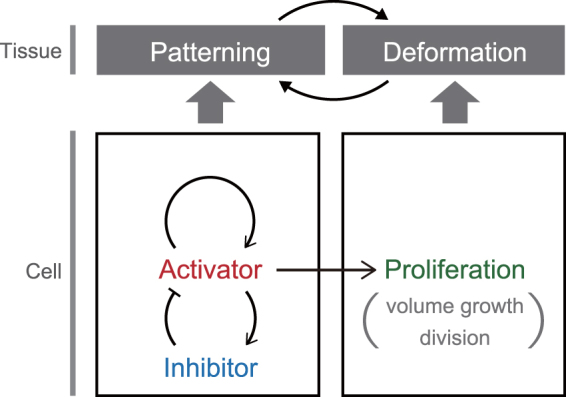
Example of modeling cell mechanical and chemical behaviors; model of cell behaviors with growth regulation on an activator–inhibitor system. The activator acts as a mitogen.

To investigate what can emerge from the coupling of multicellular patterning and deformation, we simulated the tissue growth dynamics regulated by an activator–inhibitor system (Fig. 2). Previous studies have shown that the activator-inhibitor process operates growth regions in the 3D tissue structure^13^. For this simulation, we assumed that tissue deformations are simply driven by the mechanical forces generated by cell proliferation (volume growth and division), whose rate has a positive dependence on the intracellular concentration of activator molecules, represented by ${\mathscr{A}}$. Hence, the activator molecules act as a mitogen in this system. Moreover, the activator molecules have a negative feedback through inhibitor molecules, represented by ${\mathcal I}$. Details of the mathematical assumption are described below.

### Viscoelastic behaviors of cells

The elastic properties of cells are expressed by the effective energy *U* in Eq. (1) as follows:

3 $$U=\sum _{j}^{{\rm{cell}}}\frac{1}{2}{k}_{{\rm{v}}}{(\frac{{v}_{j}}{{v}_{{\rm{eq}}j}}-1)}^{2}+\sum _{j}^{{\rm{cell}}}{\kappa }_{{\rm{s}}}{s}_{j}$$

On the right hand side of Eq. (3), the first and second terms are the volume elastic energy and surface energy of the *j*th cell, respectively. Constants *k*_v_ and *κ*_s_ are the volume elasticity and surface energy, respectively. Variables *v*_*j*_ and *s*_*j*_ are the current volume and surface area of the *j*th cell, respectively. Variable *v*_eq*j*_ is an equilibrium volume of the *j*th cell.

For simplification, we assume that the viscous property is homogeneous in the entire tissue by ignoring the inhomogeneity of tissue structures such as apical actin belts and basement membrane. Hence, viscous property of cells is simply expressed using functions of *η*_*i*_ and ***V***_f*i*_ in Eq. (1) as employed in our previous study^32^. In this model, friction *η*_*i*_ is defined as the summation for the all surrounding cells:

4 $${\eta }_{i}=\sum _{j}^{{\rm{cell}}}{\eta }_{{\rm{c}}},$$

where constant *η*_c_ reflects a viscous friction of vertices from the surrounding cell. Velocity ***V***_f*i*_ is defined as the average velocity of the surrounding cells, where a velocity of the *j*th cell is defined as the average velocity of the vertices composing the *j*th cell.

### Activator–inhibitor system

As an example of the Turing model, we employed the activator–inhibitor system suggested by Gierer and Meinhardt^37^. To focus on the coupling of patterning and deformation, we simplify the equations of activator and inhibitor dynamics in a discrete manner. Dynamics of the numbers of activator and inhibitor molecules within the *j*th cell, represented by ${m}_{{\mathscr{A}}j}$ and ${m}_{ {\mathcal I} j}$, are expressed as follows:

5 $$\frac{\partial {m}_{{\mathscr{A}}j}}{\partial t}={\mu }_{{\mathscr{A}}}\sum _{k(j)}^{{\rm{cell}}}(\frac{{m}_{{\mathscr{A}}k}}{{v}_{k}}-\frac{{m}_{{\mathscr{A}}j}}{{v}_{j}}){s}_{jk}^{{\rm{cc}}}+\frac{\gamma {({m}_{{\mathscr{A}}j})}^{2}}{{m}_{ {\mathcal I} j}}-\gamma {m}_{{\mathscr{A}}j},$$

6 $$\frac{\partial {m}_{ {\mathcal I} j}}{\partial t}={\mu }_{ {\mathcal I} }\sum _{k(j)}^{{\rm{cell}}}(\frac{{m}_{ {\mathcal I} k}}{{v}_{k}}-\frac{{m}_{ {\mathcal I} j}}{{v}_{j}}){s}_{jk}^{{\rm{cc}}}+\frac{\gamma {({m}_{{\mathscr{A}}j})}^{2}}{{\rho }_{{\rm{u}}}{v}_{j}}-\gamma {m}_{ {\mathcal I} j}\mathrm{.}$$

The first terms in the right hand side of Eqs (5) and (6) are flux, where constants ${{\mu }}_{{\mathscr{A}}}$ and ${{\mu }}_{ {\mathcal I} }$ are the transport coefficients of activator and inhibitor molecules, respectively. The second and third terms in the right hand side of Eqs (5) and (6) are production and degradation terms, where constant *γ* is the reaction rate of the activator and inhibitor molecules and constant *ρ*_u_ is the reference concentration. The discrete formulation of Eqs (5) and (6) corresponds to the continuum formulation described in Sect. 7 in Appendix.

### Mechanochemical coupling

To couple multicellular deformation with patterning, we introduce cell proliferation depending on the activator concentration. During cell proliferation, the equilibrium volume *v*_eq*j*_ increases depending on the concentration of activator molecules, ${m}_{{\mathscr{A}}j}/{v}_{j}$. Moreover, when *v*_eq*j*_ increases up to *v*_th_, the *j*th cell divides into two daughter cells with (1/2)*v*_th_. Constant *v*_th_ is set to be (4/3)*v*_ref_ as if the median of all *v*_eq*j*_ is around *v*_ref_.

In biological systems, the cell growth rate is regulated by the concentration of mitogen. Hence, by regarding the activator as a mitogen, the growth rate of the *j*th cell volume, represented by ${\dot{v}}_{{\rm{eq}}j}={\rm{d}}{v}_{{\rm{eq}}j}/{\rm{d}}t$, is defined as

7 $${\dot{v}}_{{\rm{eq}}j}={\lambda }_{j}{v}_{{\rm{ref}}},$$

8 $${\lambda }_{j}=\frac{{({m}_{{\mathscr{A}}j}/{v}_{j})}^{\alpha }}{{({\rho }_{{\rm{sw}}})}^{\alpha }+{({m}_{{\mathscr{A}}j}/{v}_{j})}^{\alpha }}{\lambda }_{{\rm{ref}}j},$$

which is the Hill equation where *α* is the Hill coefficient. Scalar *λ*_*j*_ is the temporal growth rate of the *j*th cell. Constant *ρ*_sw_ is the switching concentration of the activator molecules. Scalar *λ*_ref *j*_ is the temporal reference growth rate of the *j*th cell, which is randomly given to follow a Gaussian distribution. By solving the first time passage of the cell volume growth from (1/2)*v*_th_ to *v*_th_, the mean and variance of the Gaussian distribution are uniquely determined. Thereby, in the case of ${m}_{{\mathscr{A}}j}/{v}_{j}\gg {\rho }_{{\rm{sw}}}$, cell cycle periods are distributed according to an inverse Gaussian distribution of mean *τ*_cycle_ and standard deviation *τ*_sd_. Here, constant *τ*_sd_ is empirically determined as 0.1*τ*_cycle_.

Cell division is represented by dividing a single polyhedron at a plane; the direction of the dividing plane is regulated to be normal to the longest axis of the individual cell shape, on the plane of the tissue surface. Details of the cell division manner are similar to those of the local regulation used in our previous studies^30,38^. In the division process, morphogen concentrations in daughter cells are set to be the same values with those of the mother cell.

### Physical parameter setting

To focus on the coupling of patterning and deformation, we simplified Eqs (5) and (6) by introducing two parameters *χ* and *ϕ*. Constants *χ* and *ϕ* express spatial and temporal characteristics, respectively, and were defined as functions of ${{\mu }}_{{\mathscr{A}}}$, ${{\mu }}_{ {\mathcal I} }$, and *γ* as *χ* = ${{\mu }}_{{\mathscr{A}}}/{{\mu }}_{ {\mathcal I} }$ and *ϕ* = ${{\mu }}_{ {\mathcal I} }/\gamma$. Using *χ* and *ϕ*, Eqs (5) and (6) were rewritten as

9 $$\frac{\partial {m}_{{\mathscr{A}}j}}{\partial t}=\gamma (\chi {\varphi }\sum _{k(j)}^{{\rm{cell}}}(\frac{{m}_{{\mathscr{A}}k}}{{v}_{k}}-\frac{{m}_{{\mathscr{A}}j}}{{v}_{j}}){s}_{jk}^{{\rm{cc}}}+\frac{{({m}_{{\mathscr{A}}j})}^{2}}{{m}_{ {\mathcal I} j}}-{m}_{{\mathscr{A}}j}),$$

10 $$\frac{\partial {m}_{ {\mathcal I} j}}{\partial t}=\gamma ({\varphi }\sum _{k(j)}^{{\rm{cell}}}(\frac{{m}_{ {\mathcal I} k}}{{v}_{k}}-\frac{{m}_{ {\mathcal I} j}}{{v}_{j}}){s}_{jk}^{{\rm{cc}}}+\frac{{({m}_{{\mathscr{A}}j})}^{2}}{{\rho }_{{\rm{u}}}{v}_{j}}-{m}_{ {\mathcal I} j})\mathrm{.}$$

Under the steady state, the terms in the left sides of Eqs (9) and (10) become zero. Therefore, steady spatial patterns can be determined by *χ* independent on *γ*, and the time scale of the patterning can be regulated by *γ* while maintaining the same steady pattern. Based on the linear approximation, the length scale of steady activator patterns is proportional to *χ*^1/4^*ϕ*^1/2^.

To solve Eqs (1, 9 and 10), parameter values were normalized by unit length (*v*_ref_)^1/3^, unit number of molecules *ρ*_u_*v*_ref_, unit energy 5*κ*_s_(*v*_ref_)^2/3^, and unit time $4{\eta }_{{\rm{c}}}\mathrm{/5}{\kappa }_{{\rm{s}}}$. Hence, normalized values of several physical parameters are given as *v*_ref_ = 1, *ρ*_u_ = 1, *κ*_s_ = 0.2, and *η*_c_ = 0.25. Hereafter, physical parameters were described as dimensionless values.

Physical parameters are summarized in Table 1. By assuming an incompressibility, the cell volume elasticity *k*_v_ was set to be much larger than the characteristic surface energy of a cell, represented by *κ*_s_(*v*_ref_)^2/3^. By assuming a quasi-static process, the cell cycle *τ*_cycle_ was set to be much larger than the characteristic time of cell deformation, represented by *η*_c_/*κ*_s_. Moreover, to focus on the coupling phenomena, the physical parameters of the patterning and coupling were fixed; because the steady-state solutions of Eqs (9) and (10) are $({m}_{{\mathscr{A}}}^{\ast 1},{m}_{ {\mathcal I} }^{\ast 1})=(0,\,0)$ and $({m}_{{\mathscr{A}}}^{\ast 2},{m}_{ {\mathcal I} }^{\ast 2})=({\rho }_{{\rm{u}}}{v}_{{\rm{ref}}},{\rho }_{{\rm{u}}}{v}_{{\rm{ref}}})$, the switching concentration for cell growth was simply set to be the medial value of the steady state solutions ${\rho }_{{\rm{sw}}}=({m}_{{\mathscr{A}}}^{\ast 1}+{m}_{{\mathscr{A}}}^{\ast 2})\mathrm{/2}{v}_{{\rm{ref}}}$. The Hill coefficient *α* was set to be much larger than unit to approximate Eq. (8) as a step-wise function. The diffusivity of inhibitor that determines the distance between activator domains was simply set to be the square of the length scale of 3 cells (*ϕ* ≈ (3(*v*_ref_)^1/3^)^2^/(4*η*_c_/5*κ*_s_)). Hence, the patterning and deformation behaviors in this model are determined by two physical parameters *γ* and *χ*. Numerical implementation is described in Sect. 8 in Appendix.

**Table 1:** Physical parameters.

Symbol	Description	Value	Basis	Eq.
*k*_v_	Cell volume elasticity	10	$\gg {\kappa }_{{\rm{s}}}{({v}_{{\rm{ref}}})}^{\frac{2}{3}}$	(3)
*κ*_s_	Cell surface energy	0.2	To define units	(3)
*η*_c_	Friction coefficient	0.25	To define units	(4)
*v*_ref_	Reference cell volume	1	To define units	(7)
*τ*_cycle_	Cell cycle	50	$\gg {\eta }_{{\rm{c}}}/{\kappa }_{{\rm{s}}}$	(7)
*ρ*_sw_	Switching concentration for growth rate	0.5	$({m}_{{\mathscr{A}}}^{\ast 1}+{m}_{{\mathscr{A}}}^{\ast 2})\mathrm{/2}{v}_{{\rm{ref}}}$	(8)
*α*	Hill coefficient in growth rate	10	$\gg 1$	(8)
*ρ*_u_	Reference morphogen concentration	1	To define units	(9, 10)
*ϕ*	Diffusivity of inhibitor	10	${(3{({v}_{{\rm{ref}}})}^{\frac{1}{3}})}^{2}/(4{{\eta }}_{{\rm{c}}}/5{{\kappa }}_{s})$	(9, 10)
*γ*	Time characteristics of patterning	0.01–100	Varied	(9, 10)
*χ*	Spatial characteristics of patterning	0.001–0.1	Varied	(9, 10)

## Results

### Turing patterns in 3D cell aggregates

First, to test whether the model can create the periodic pattern of the activator–inhibitor system in 3D cell aggregates, we simulated patterning processes while without cell volume growth (${\dot{v}}_{{\rm{eq}}j}=0$ in Eq. (7)). The arrested tissue morphologies were simply set to be a monolayer cell sheet and a compacted cell aggregate. These tissues are composed of about 200 cells, and the initial molecular concentrations were randomly set to satisfy the mean values $\langle {m}_{{\mathscr{A}}j}/{v}_{j}\rangle$ = 0.5 and $\langle {m}_{ {\mathcal I} j}/{v}_{j}\rangle$ = 0.1. Physical parameters were simply set to be *χ* = 0.1 in case of the monolayer cell sheet and *χ* = 0.05 in case of the compacted cell aggregate.

**Figure 3:**
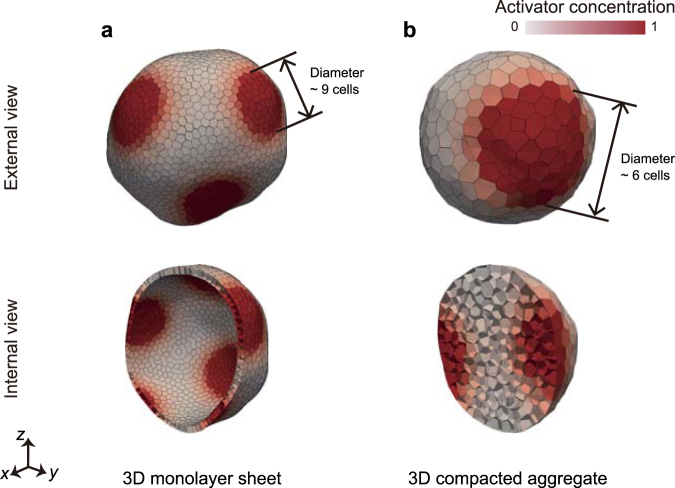
Example of chemical patterns in 3D cell aggregates, obtained by combining the Turing and 3D vertex models. (**a**) External and internal views of a monolayer cell sheet, under a condition without tissue deformations. (**b**) External and internal views of a compacted cell aggregate, under a condition without tissue deformations. Note that the magnifications of images are different between (**a** and **b)**. In (**a** and **b)**, cells are colored by activator concentration (red). To obtain the tissue in **a**, physical parameters were set as *χ* = 0.1. To obtain the tissue in (**b)**, physical parameters were set as *χ* = 0.05.

Figure 3 shows the steady patterns generated in the 3D cell aggregates, which were driven by the activator–inhibitor system. In case of the monolayer cell sheet, in-plane spot patterns were generated on the sheet (Fig. 3a). In case of the compacted cell aggregate, 3D spot patterns were generated inside the aggregate (Fig. 3b). While the patterning in 2D space has been well studied, the research of the patterning in 3D space has been just started^7,8^. To focus on the coupling phenomena, thereby, we used a monolayer cell sheet as initial tissue morphologies for the analyses.

### Patterning hysteresis on deforming tissues

Second, to analyze directional effects from deformation to patterning, we simulated patterning processes on the tissues that grow independently on the molecular concentration. To express cell growth that is independent on the molecular concentration, cell growth rate was set to be constant as *λ*_*j*_ = *λ*_ref *j*_ in Eq. (8). Moreover, because the 2D spatial patterns driven by the Turing model has been well studied, constant *χ* that determines the spatial scale of the patterning was fixed as *χ* = 0.1 and constant *γ* that determines the time scale of the patterning was varied. Here, the initial tissue morphology was simply set to be a spherical vesicle of a monolayer cell sheet composed of about 200 cells, and the initial tissue patterns were randomly set to satisfy the mean values $\langle {m}_{{\mathscr{A}}j}/{v}_{j}\rangle$ = 0.5 and $\langle {m}_{ {\mathcal I} j}/{v}_{j}\rangle$ = 0.1.

**Figure 4:**
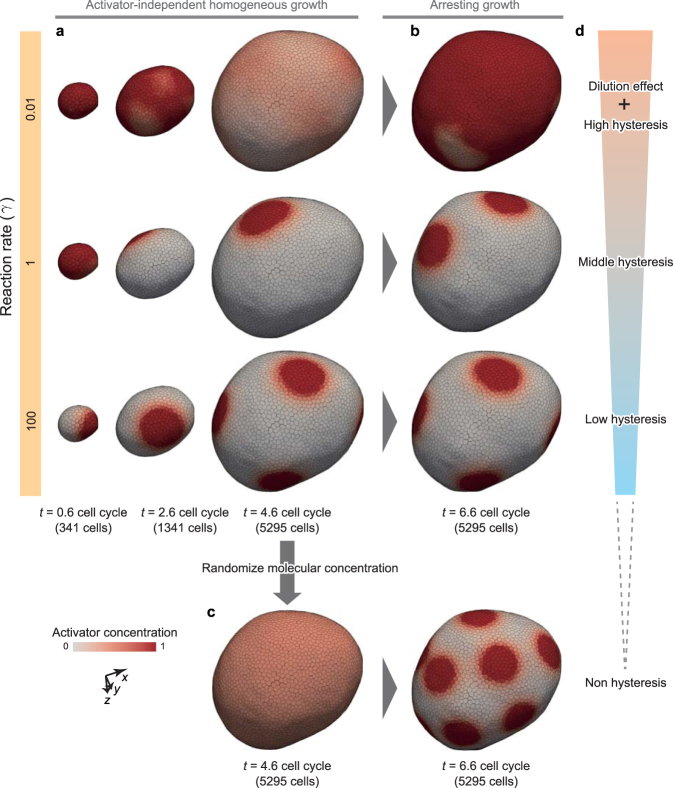
Hysteresis of patterning in deformation process. (**a**) Time series images of tissue patterning under the condition of activator-independent homogeneous cell growth. (**b**) Developed pattern without cell growth on the arrested tissue resulting from the simulation at *t* = 4.6 cell cycles in (**a**). (**c**) Regenerated pattern on the arrested tissue without cell growth, after homogenizing the molecular concentration at *t* = 4.6 cell cycles. In (**a** and **b**), cells are colored by their activator concentration. (**d**) Categorization of phenomena based on the difference in time scales between patterning and deformation. To obtain the tissues in (**a**), physical parameters were set as *χ* = 0.1. To obtain the tissues in (**b**,**c**), cell growth was arrested, and physical parameters were set as *χ* = 0.1.

As described in Eqs (9) and (10), steady spatial patterns are independent on *γ*. Consistently, when *γ* = 1 and 100 (the rate of patterning is faster than or equal to that of deformation), steady patterns were formed as shown in Fig. 4a. In this case, the activator domain size is almost independent on *γ*, and is almost maintained during tissue growth. Interestingly, the number of activator spots was maintained in the case of *γ* = 1, whereas it increased in the case of *γ* = 100. Moreover, when *γ* = 0.01 (the rate of patterning is much slower than that of deformation), steady patterns were not formed, and activator concentration was gradually blurred (Fig. 4a). This was most likely caused by the dilution effect resulting from cell growth.

To clarify whether the patterns at *t* = 4.6 cell cycles in Fig. 4a reached steady states, we simulated patterning processes on the tissues with cell growth upto *t* = 4.6 (as in Fig. 4a) and without cell volume growth after *t* = 4.6. Figure 4b shows patterns of the tissues at *t* = 6.6 cell cycles. In the case where *γ* = 0.01, activator concentrations increased in the whole of the tissue. In the case where *γ* = 1 and 100, the numbers of activator spots were similar to those at *t* = 4.6 cell cycles. These results suggest that the patterning shows the dependence on its history, i.e., hysteresis, which becomes weaker in case with higher *γ*.

Moreover, we also randomize the molecular distribution on the grown tissue at *t* = 4.6 cell cycles in Fig. 4a, and simulated a repatterning process while arresting cell volume growth, as shown in Fig. 4c. Here, the tissue pattern was randomly set to satisfy the mean values $\langle {m}_{{\mathscr{A}}j}/{v}_{j}\rangle$ = 0.5 and $\langle {m}_{ {\mathcal I} j}/{v}_{j}\rangle$ = 0.1. As a result, a new pattern emerged on the tissue, in which the number of spots is greater than that generated on the growing tissues in Fig. 4b. These results clarified that the hysteresis becomes weaker in case with higher *γ*. Interestingly, even when increasing *γ* greatly, the number of spots on the growing tissue (*γ* = 0.01 in Fig. 4a) is still smaller than that on the arrested tissue (Fig. 4c). Hence, it seems difficult to completely suppress the hysteresis of patterning by regulating *γ*.

The dependence of the patterning on *γ* can be understood by the ratio of time scales of patterning to deformation (Fig. 4d). Because the rate of patterning is limited by the reaction rate, it can be affected by *γ*. On the other hand, because the deformation rate is limited by the rate of cell growth, it can be affected by 1/*τ*_cycle_. Where the patterning rate *γ* is similar to that of deformation 1/*τ*_cycle_, patterning shows high hysteresis in addition to a dilution effect in that initial molecular concentrations are strongly maintained and gradually blurred by the dilution effect of cell volume growth (*γ* = 0.01 in Fig. 4a,d). Where the patterning rate *γ* is slightly larger than that of deformation 1/*τ*_cycle_, patterning shows middle hysteresis in that it is rapid enough to maintain the existing domain areas and slow enough to maintain their positions on tissue (*γ* = 1 in Fig. 4a,d). Where the patterning rate *γ* is much larger than that of deformation 1/*τ*_cycle_, patterning shows low hysteresis; it is rapid enough to change the existing domain areas, but is too fast to keep their positions on tissue (*γ* = 100 in Fig. 4a,d). These results highlight the importance of the difference in time scales between patterning and deformation.

### Coupling patterning and deformation drives undulation, tubulation, and branching

Third, we investigate the coupling phenomena of patterning and deformation. The initial tissue morphology was simply set to be a spherical vesicle of a monolayer cell sheet composed of about 2,000 cells, whose patterns reached steady states. The steady patterns depend on *χ*; the model provided various tissue deformations, and several typical deformations such as undulation, tubulation, and branching were shown.

**Figure 5:**
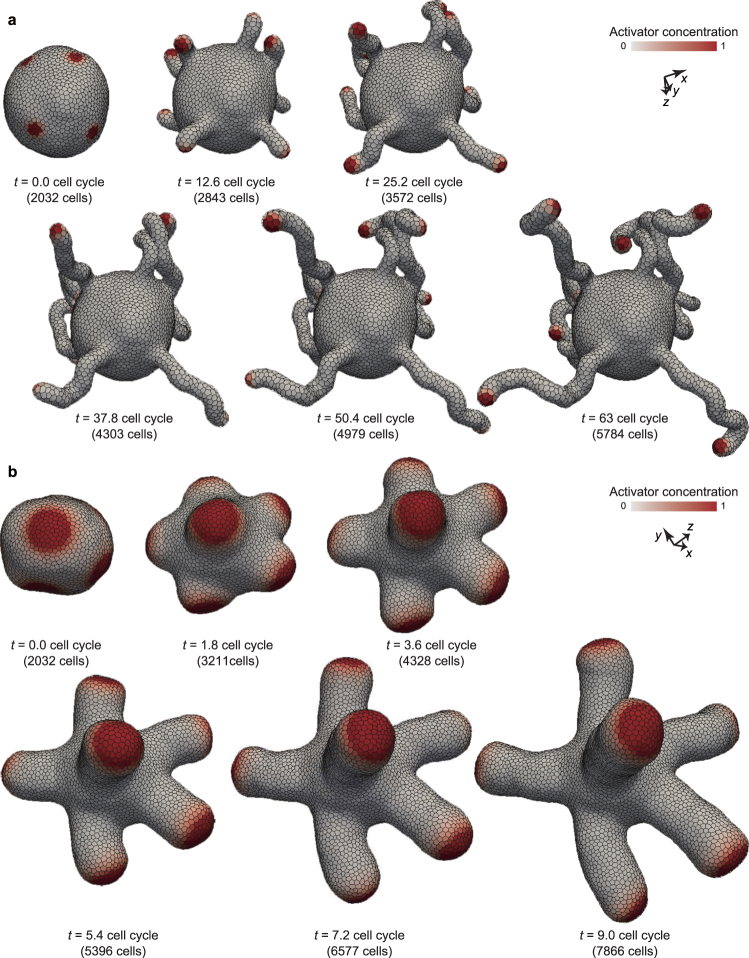
Tubulation. (**a**) Time series images of thin tube formation. (**b**) Time series images of thick tube formation. In (**a** and **b**), cells are colored by their activator concentration. These deformation processes are also shown in Supplementary Movies 1 and 2. In (**a**), physical parameters were as *χ* = 0.01 and *γ* = 100. In (**b**), physical parameters were as *χ* = 0.1 and *γ* = 1.

Figure 5 shows tubulation deformation processes under the conditions with *χ* = 0.01 and *χ* = 0.1, where several bumps sprouted from steady domains of activator regions on the vesicle, and grew into tubes. During tubulation, the activator molecules stayed around the tip of tubes, from which the tubes continuously grew.

As described in Sect. 3.4, the size of activator regions should be approximately proportional to *χ*^1/4^*ϕ*^1/2^; hence, the size in the case of *χ* = 0.1 is expected to be about 1.8 times larger than that in the case of *χ* = 0.01. According to the expectation, the tube diameter varied between thin (*χ* = 0.01 in Fig. 5a) and thick (*χ* = 0.1 in Fig. 5b). Moreover, because the size of the activator spots is maintained, the tube diameters are constant under the individual conditions. Since the bending rigidity of tubes depends on their diameters, increasing *χ* leads to straightening the tubes; while thin tubes became undulated (*χ* = 0.01 in Fig. 5a), thick tubes became straight (*χ* = 0.1 in Fig. 5b).

**Figure 6:**
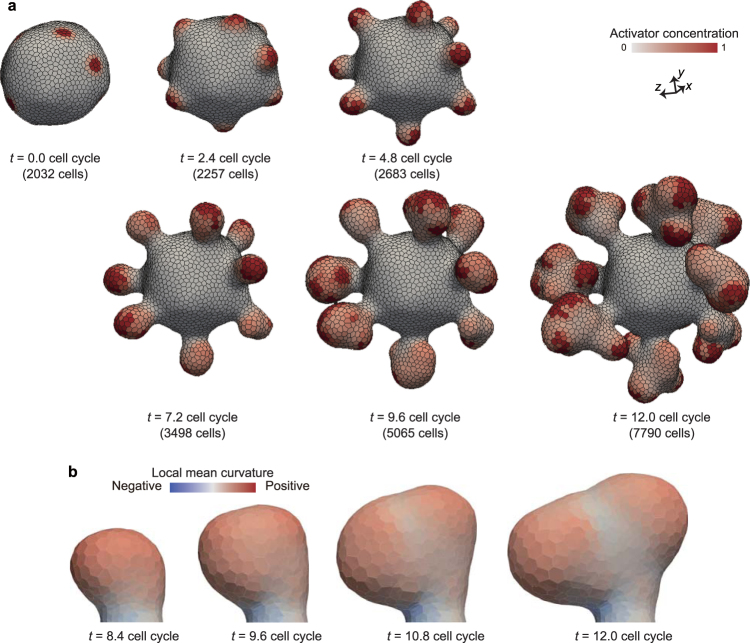
Branching. (**a**) Time series images of whole tissue deformation. Cells are colored by local mean curvature. The deformation process is also shown in Supplementary Movie 3. Physical parameters were set as *χ* = 0.01 and *γ* = 0.01. (**b**) Time series images of branch structure.

Figure 6 shows a branching deformation process, in which several spherical protrusions sprouted from steady domains of activator regions on the vesicle (Fig. 6a), and separated into multiple bumps (Fig. 6b). Interestingly, the molecular concentration was distortedly randomized in the sprouted region as shown in Fig. 6a. This is because of the discrete description of multicellular structure; the activator concentration tends to relax into equilibrium within individual cell compartments before diffusing to the surrounding cells. This non-cooperative behavior of cells tend to form discontinuous molecular distributions in space, which facilitate the domain branching as a perturbation. Therefore, the mechanism of this branching deformation seems partially different from those expected from the domain branching in the continuum space, where molecular concentrations tend to be continuously distributed in space.

**Figure 7:**
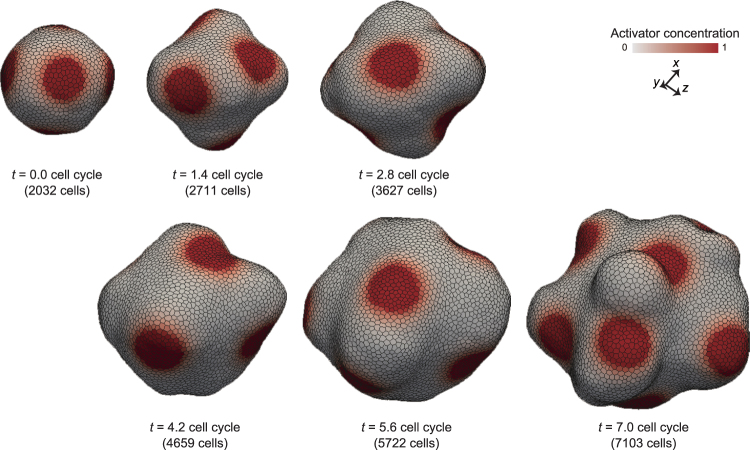
Undulation. Time series images of whole tissue deformation. Cells are colored by their activator concentration. The deformation process is also shown in Supplementary Movie 4. Physical parameters were set as *χ* = 0.1 and *γ* = 100.

Figure 7 shows an undulation deformation process, where activator domains dynamically move on the tissue. The pattern can be dynamically remodeled by the disturbance of cell growth. In Figs 5 and 6, the pattern remodeling is much slower than the tissue growth. On the other hand, in Fig. 7, the pattern remodeling is much faster than the tissue growth, and the activator domains rapidly move before forming some protrusions. These results suggest that the coupling of patterning and deformation creates a greater variation of dynamics than expected.

### Difference in time scales between patterning and deformation varies a 3D morphology of growing tissues

**Figure 8:**
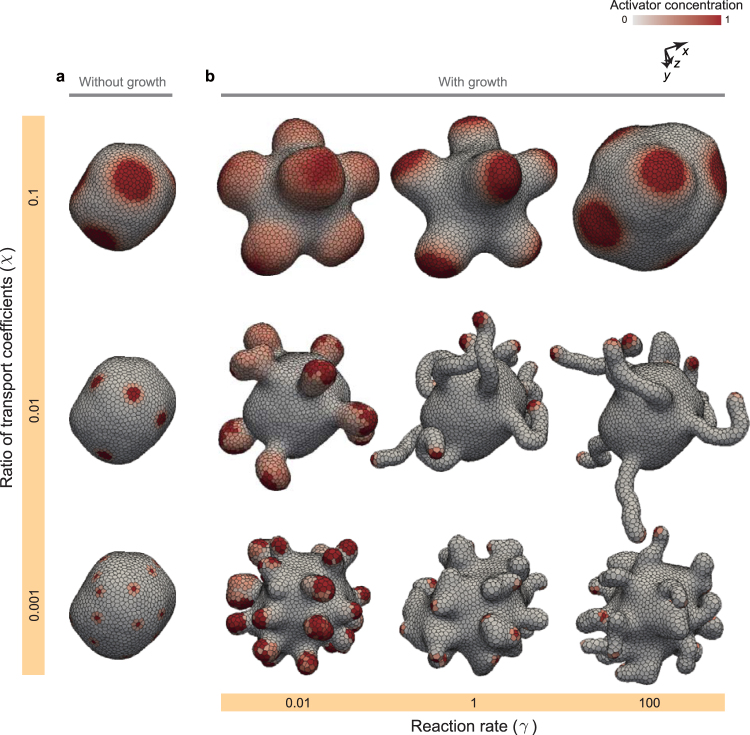
Multiple unstable mode of activator–inhibitor system in one-dimensional space. (**a**) Dispersion relation of the activator–inhibitor system. Growth speed of each wavenumber component is positive in the region of 4.0 ≤ *k* ≤ 8.5. Dashed lines indicate the possible wavenumber components. (**b**) Example results of the numerical simulations for the activator–inhibitor system with respect to the different initial conditions. The initial conditions were set as a random distribution with the uniform white noise, the boundary condition was set as ${{\rho }}_{{\mathscr{A}}}$ = ${{\rho }}_{ {\mathcal I} }$ = 1 at *x* = 0, 2*π*, and physical parameters were set as ${D}_{{\mathscr{A}}}/\gamma$ = 0.01 and ${D}_{ {\mathcal I} }/\gamma$ = 0.1. Red and blue lines indicate activator and inhibitor, respectively. (**c**) Result of the numerical simulation from the initial condition with dominant number of waves 4. (**d**) Result of the numerical simulation with domain growth from the initial condition with dominant number of waves 4. The domain size grew from 2*π* to 4*π*. In (**c** and **d**), the initial condition was set as periodic as ${{\rho }}_{{\mathscr{A}}}$ = ${{\rho }}_{ {\mathcal I} }$ = 1 + sin4*x*.

Fourthly, to understand the general behaviors of tissue patterning and deformation, we calculated the morphology diagram with respect to two physical parameters: *χ* and *γ* (Fig. 8). The initial tissue morphology was simply set to be a spherical vesicle of a monolayer cell sheet composed of about 2,000 cells, whose patterns reached steady states. The steady patterns depend on *χ* as shown in Fig. 8a; with increasing *χ*, the size of spots increased and the number of spots decreased.

**Figure 9:**
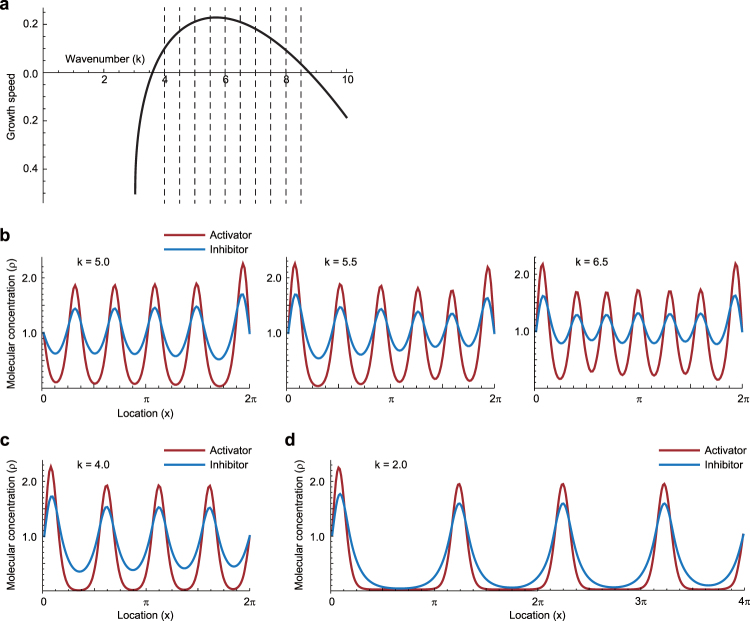
Morphology diagram. (**a**) Images of tissue patterns obtained by simulations without cell growth. (**b**) Morphology diagram of tissue shapes and patterns obtained by simulations with cell growth. In (**a** and **b**), cells are colored by their activator concentration. The steady tissue patterns in (**a)** were obtained by simulations during 2 cell cycles, in which physical parameters were set as *γ* = 100. The individual tissues in (**b**) were composed of about 4,000 cells, which were picked up on the growth process.

Figure 8b shows a morphology diagram of tissue patterns and deformations with respect to *χ* and *γ*. Importantly, by comparing the tissues which have the same value of *χ*, the tissue patterns in Fig. 8b were partially different from the arrested tissues in Fig. 8a, and the tissue morphologies in Fig. 8b varied by *γ*. For example, the molecular distribution within activator domains where *γ* = 0.01 in Fig. 8b, was inhomogeneous and more blurred than those of the arrested tissues in Fig.8a. In the case of *χ* = 0.1, while the tissues where *γ* = 0.01 and 1 formed thick tube structures, the tissue in case with *γ* = 100 was undulated. In the case of *χ* = 0.01, the tissue where *γ* = 0.01 formed spherical protrusions, while the tissues where *γ* = 1 and 100 formed thin tube structures.

The dependence of the tissue morphologies on *γ* can be understood by the hysteresis in patterning. Where patterning shows high hysteresis (*γ* = 0.01), thereby there tend to remain a disturbance in molecular distribution by cell growth and division at a single cell level, which repatterns activator domains to be separated to form branch structures (Fig. 6). Where patterning shows middle hysteresis (*γ* = 1) in that it maintains the existing domain areas and their positions on tissue; thus, the middle hysteresis causes the tube structures (Fig. 5). Where patterning shows low hysteresis (*γ* = 100); it dynamically changes positions of domain areas on tissue; thus, the low hysteresis causes the undulation structures (Fig. 7). These results suggest that the tissue morphologies emerging from the coupling phenomena depend on both spatial and temporal properties of patterning.

### Hysteresis in patterning emerges from the multiple unstable mode in activator–inhibitor system

As shown in Figs 4–8, the computational simulations combining the Turing and 3D vertex models revealed that the patterning hysteresis partially plays an important role in tissue formations emerging from the coupling phenomena. The patterning hysteresis has been implicitly reported in the Turing pattern resulting from a simple one-dimensional model with growing domain^39^; under the quasi-static process of domain growth, the wavelength of the pattern can change without increasing the number of spots. Lastly, to understand the patterning hysteresis more clearly, we performed the linear stability analysis of the continuum type of the activator–inhibitor system, described in Sect. 7 in Appendix, in one-dimensional space (0 ≤ *x* ≤ 2*π*). The analysis revealed that this model has multiple unstable modes in dispersion relation as shown in Fig. 9a; and this system had the most unstable wavenumber, represented by *k*_max_ (around 5.5 in this case). Hence, we expect that the wavenumber, represented by *k*, varies around *k*_max_.

To clarify the variation of the wavenumber, we performed the numerical simulation of the continuum type of the activator–inhibitor system, described in Sect. 7 in Appendix, in one-dimensional space (0 ≤ *x* ≤ 2*π*). As expected above, when the initial condition of ${{\rho }}_{{\mathscr{A}}}$ and ${{\rho }}_{ {\mathcal I} }$ was set as a random distribution with the uniform white noise, the resulting *k* under the steady state varied from 5.0 to 6.5 depending on the initial condition as shown in Fig. 9b. More interestingly, when the initial condition was set as a periodic distribution with *k* = 4.0, the resulting *k* became 4.0 as shown in Fig. 9c, which is different from those resulting from the initial condition with a random distribution in Fig. 9b.

Moreover, to understand the effect of domain growth on patterning, we additionally took into account the domain growth in the above simulation. We set *L* to be 2*π* under the initial condition with *k* = 4.0. Then, we calculated the molecular distribution after expanding *L* to be 4*π* slowly enough to satisfy the quasi-static process. As a result, even when *k* became 2.0 that is out of the unstable region of the dispersion relation in Fig. 9a, the initial number of waves was robustly maintained as shown in Fig. 9d. Thus, the patterning can vary depending on the initial condition and show hysteresis in the process of domain growth.

## Discussion

Chemical patterning plays a role in regionalizing a tissue, in which a higher-order structure is constructed by integrating different functional cells. The patterning has been simply explained by the Turing model^4^, and observed in biological systems^5^. Despite the difficulty in observing molecular diffusions *in vivo*, the Turing model has had a significant impact on the understanding of embryonic morphogenesis. Numerical studies over the decades have suggested various kinds of patterns in 2D space^37^, and have led to discovery of further complex patterns in 3D space^7,8^. Currently, descriptions using integral kernel equations have been suggested to explain biological patterns more generally^40^. Moreover, recent studies using continuum models have emphasized the importance of the coupling between patterning and deformation in 3D space^9^. Based on this knowledge, this study has demonstrated computational simulations of 3D multicellular deformation coupled with patterning in the single cell level, which may help connect these basic findings to the understanding of biological systems.

In this study, by combining the Turing and 3D vertex models, a reaction–diffusion process was expressed simply by an activator–inhibitor system coupled with cell growth. The simple coupling system caused various large tissue deformations, which highlighted the importance of the difference in time scales between patterning and deformation. Importantly, branch structures were caused by large tissue deformations, and the inhomogeneous molecular distribution within activator domains was caused by the dilution effect on discrete cells. Therefore, the findings in this study were due to the combined model which describes mechanical and chemical behaviors in the single cell level.

Because of the 3D description, the proposed model can be applied not only to a monolayer cell sheet, but also to a compacted cell aggregate. Moreover, the proposed model can be also applied to the tissue dynamics that can be affected by individual cell deformations; in this study, the deformations at the single cell level have little effects on tissue dynamics since we assumed the homogeneous, isotropic property for cell mechanical behaviors as in Eq. (3). On the other hand, if we assume inhomogeneous and/or anisotropic property, morphogen patterns would be largely distorted in the tissue level via the flux in Eq. (2). Because of the cell-based description of the model, the method can be applied to the complex phenomena of cell mechanical behaviors (deformation, rearrangement, division, and apoptosis) with chemical behaviors (e.g., differentiation and growth) at the single cell level. Such a cell-based 3D description is useful in the analysis of the complex phenomena of multicellular patterning and deformation. Notably, the applicable area of the method is limited on a single cell level; intracellular patterning can be expressed over a length scale of cell–cell boundaries, which is a unit length of vertex models. The method therefore has the potential for remarkable predictions of undiscovered multicellular dynamics at a single cell level, which could guide future experimental approaches.

In summary, we demonstrated the computational simulation of multicellular deformations, coupled with reaction–diffusion processes in 3D space. As an example, we showed that coupling an activator–inhibitor system with cell growth caused various tissue deformations such as undulation, tubulation, and branching; the cell–based 3D analyses led to discovery of the importance of the time scales of patterning and deformation. The model has a significant applicability to 3D multicellular dynamics, compounding mechanical and chemical cell behaviors at a single cell level. Thus, we anticipate the combined model to open up a new avenue of elucidating yet undiscovered phenomena emerging from mechanochemical coupling in multicellular dynamics.

### Appendix A: Relationship between discrete and continuum Turing models

While the proposed model combined the discrete Turing with 3D vertex models, the Turing model is often described in a continuum manner. To understand the relationship between the discrete and continuum Turing models, we derive the discrete model from the continuum model.

Here, we introduce the ${\mathcal M}$th molecular concentration as a function of an arbitrary location vector, represented by ${{\rho }}_{ {\mathcal M} }({\boldsymbol{x}})$. The dynamics of ${{\rho }}_{ {\mathcal M} }$ is generally described by

A1 $$\frac{\partial {\rho }_{ {\mathcal M} }({\boldsymbol{x}})}{\partial t}={\boldsymbol{\nabla }}\cdot ({D}_{ {\mathcal M} }{\boldsymbol{\nabla }}{\rho }_{ {\mathcal M} }({\boldsymbol{x}}))+{R}_{ {\mathcal M} }({\{{\rho }_{ {\mathcal M} }({\boldsymbol{x}})\}}_{ {\mathcal M} })\mathrm{.}$$

In Eq. (A1), the first term in the right hand side is a flux of the ${\mathcal M}$th molecules, where ${D}_{ {\mathcal M} }$ is a diffusion coefficient of the ${\mathcal M}$th molecules. The second term in the right hand side is a reaction of the ${\mathcal M}$th molecules, where ${R}_{ {\mathcal M} }({\{{{\rho }}_{ {\mathcal M} }(x)\}}_{ {\mathcal M} })$ is a reaction rate of the ${\mathcal M}$th molecules as a function of a set of molecular concentrations at ***x***.

First, we introduce the volumetric region of the *j*th cell compartment, represented by **Φ**_*j*_. Using **Φ**_*j*_, the volume and the number of the ${\mathcal M} th$ molecules within the *j*th cell compartment are described as follows:

A2 $${v}_{j}\equiv {\int }_{{\boldsymbol{x}}\in {{\boldsymbol{\Phi }}}_{j}}{\rm{d}}{\boldsymbol{x}},$$

A3 $${m}_{ {\mathcal M} j}\equiv {\int }_{{\boldsymbol{x}}\in {{\boldsymbol{\Phi }}}_{j}}{\rho }_{ {\mathcal M} }({\boldsymbol{x}})d{x}\mathrm{.}$$

Second, the total number of molecules within individual cell compartments should be conserved unless any flux, production, and degradation, even while the cell compartments dynamically deform. Hence, we assume the mass conservation for the ${\mathcal M}$th molecules within **Φ**_*j*_, where ${m}_{ {\mathcal M} j}$ is independent on the range of **Φ**_*j*_. Based on this assumption, the integral of ${\partial }_{{\rho } {\mathcal M} }/\partial t$ in Eq. (A1) over **Φ**_*j*_ can be rewritten as

A4 $${\int }_{{\boldsymbol{x}}\in {{\boldsymbol{\Phi }}}_{j}}\frac{\partial {\rho }_{ {\mathcal M} }({\boldsymbol{x}})}{\partial t}{\rm{d}}{\boldsymbol{x}}=\frac{{\rm{d}}{m}_{ {\mathcal M} j}}{{\rm{d}}t}\mathrm{.}$$

Third, we assume that the molecular concentration is homogeneous within individual cells, where the ${\mathcal M}$th molecular concentration within **Φ**_*j*_ is described as ${m}_{ {\mathcal M} j}/{v}_{j}$. Based on this assumption, the reaction rate becomes homogeneous within the *j*th cell compartment. Hence, the integral of ${R}_{ {\mathcal M} }$ in Eq. (A1) over **Φ**_*j*_ can be rewritten as

A5 $${\int }_{{\boldsymbol{x}}\in {{\boldsymbol{\Phi }}}_{j}}{R}_{ {\mathcal M} }({\{{\rho }_{ {\mathcal M} }({\boldsymbol{x}})\}}_{ {\mathcal M} }){\rm{d}}{\boldsymbol{x}}={v}_{j}{R}_{ {\mathcal M} }({\{{m}_{ {\mathcal M} j}/{v}_{j}\}}_{ {\mathcal M} })\mathrm{.}$$

Then, using Eqs (A4) and (A5), the integral of Eq. (A1) over **Φ**_*j*_ is rewritten as

A6 $$\frac{{\rm{d}}{m}_{ {\mathcal M} j}}{{\rm{d}}t}={\int }_{{{\rm{\Omega }}}_{j}}{D}_{ {\mathcal M} }({\boldsymbol{\nabla }}{\rho }_{ {\mathcal M} })\cdot {{\boldsymbol{n}}}_{j}{\rm{d}}s+{v}_{j}{R}_{ {\mathcal M} }({\{{m}_{ {\mathcal M} j}/{v}_{j}\}}_{ {\mathcal M} }),$$

where the first term in the right hand side is the surface integral over the total surface of the *j*th cell, represented by Ω_*j*_, and ***n***_*j*_ is the outer normal vector on the *j*th cell surface. In the derivation from Eqs (A1) to (A6), the Gauss divergence theorem was employed to the first term in the right hand side.

Finally, we assume that the gap width between neighboring cells and the molecular diffusion coefficients is homogeneous^36^. Here, we introduce the molecular transport rate between neighboring cells, represented by ${{\mu }}_{ {\mathcal M} }$. Constant ${{\mu }}_{ {\mathcal M} }$ can be written as ${{\mu }}_{ {\mathcal M} }={D}_{ {\mathcal M} }/{\omega }$, where ${\omega }$ is the effective gap width between neighboring cells. Using *μ*_M_, Eq. (A6) is rewritten as Eq. (2).

Based on the discrete description in the proposed model, the resulting pattern has the following characteristics: (i) Since the size of the pattern is limited over the single cell, the intracellular structure of the pattern cannot appear. (ii) The structure of the pattern depends on the distribution of individual cell shapes; the pattern can be distorted when cells have anisotropic shapes.

### Appendix B: Continuum type of activator–inhibitor system

 

B1 $$\frac{\partial {\rho }_{{\mathscr{A}}}}{\partial t}={D}_{{\mathscr{A}}}{\rm{\Delta }}{\rho }_{{\mathscr{A}}}+\gamma \frac{{({\rho }_{{\mathscr{A}}})}^{2}}{{\rho }_{ {\mathcal I} }}-\gamma {\rho }_{{\mathscr{A}}},$$

B2 $$\frac{\partial {\rho }_{ {\mathcal I} }}{\partial t}={D}_{ {\mathcal I} }{\rm{\Delta }}{\rho }_{ {\mathcal I} }+\gamma \frac{{({\rho }_{{\mathscr{A}}})}^{2}}{{\rho }_{{\rm{u}}}}-\gamma {\rho }_{ {\mathcal I} }\mathrm{.}$$

The first terms in the right hand side of Eqs (B1) and (B2) are flux, where constants ${D}_{{\mathscr{A}}}$ and ${D}_{ {\mathcal I} }$ are the diffusion coefficients of activator and inhibitor molecules, respectively. The second and third terms in the right hand side of Eqs (B1) and (B2) are production and degradation terms.

### Appendix C: Numerical implementation

The numerical algorithm procedure is similar to those in our previous study^36^. The time integration of Eqs (1, 9 and 10) were numerically performed using the Euler method, with time steps Δ*t*_*v*_ and Δ*t*_*m*_, respectively. Eq. (1) is a self-consistent equation; it was solved by convergent calculations using the iterative method, in which the mean residual error should be under the threshold value, *E*_th_^32^. According to the reversible network reconnection model^29^, local network patterns were reconnected when each edge included in a local pattern became shorter than a threshold value, Δ*l*_th_. Trials for applying the reconnection rule were conducted for each edge and each trigonal face, at each time interval Δ*t*_*r*_. Numerical parameters are shown in Table 2.

**Table 2:** Numerical parameters.

Symbol	Value	Description
Δ*t*_v_	0.005	Integration time step of Eq. (1)
Δ*t*_m_	0.0005	Integration time step of Eqs (9) and (10)
Δ*t*_r_	1.0	Time interval of network reconnections
Δ*l*_th_	0.05	Threshold length of network reconnections
*E*_th_	10^−5^	Threshold for residual error

## Electronic supplementary material

Thin tubulation

Thick tubulation

Branching

Undulation
