# Publisher Correction: Combining Turing and 3D vertex models reproduces autonomous multicellular morphogenesis with undulation, tubulation, and branching

**DOI:** 10.1038/s41598-018-24858-2

**Published:** 2018-06-04

**Authors:** Satoru Okuda, Takashi Miura, Yasuhiro Inoue, Taiji Adachi, Mototsugu Eiraku

**Affiliations:** 1 RIKEN Center for Developmental Biology, 2-2-3 Minatojima-minamimachi, Chuo-ku, Kobe, Hyogo 650-0047 Japan; 2 PRESTO, Japan Science and Technology Agency, 4-1-8 Honcho, Kawaguchi, Saitama 332-0012 Japan; 3 Faculty of Medical Sciences, Kyushu University, 3-1-1 Maidashi, Higashi-ku, Fukuoka 812-8582 Japan; 4 Institute for Frontier Life and Medical Sciences, Kyoto University, 53 Kawahara-cho, Shogoin, Sakyo-ku, Kyoto 606-8507 Japan

Correction to: *Scientific Reports* 10.1038/s41598-018-20678-6, published online 05 February 2018

This Article contains errors in the order of the Figures and Figure Legends. In the HTML version of the Article, Figures 8 and 9 were published in the reverse order, but not their accompanying legends. In the PDF version of the Article, the legends of Figures 8 and 9 only were published in the reverse order. The correct Figures 8 and 9 together with their accompanying legends appear below as Figures 1 and 2 respectively.

**Figure 1:**
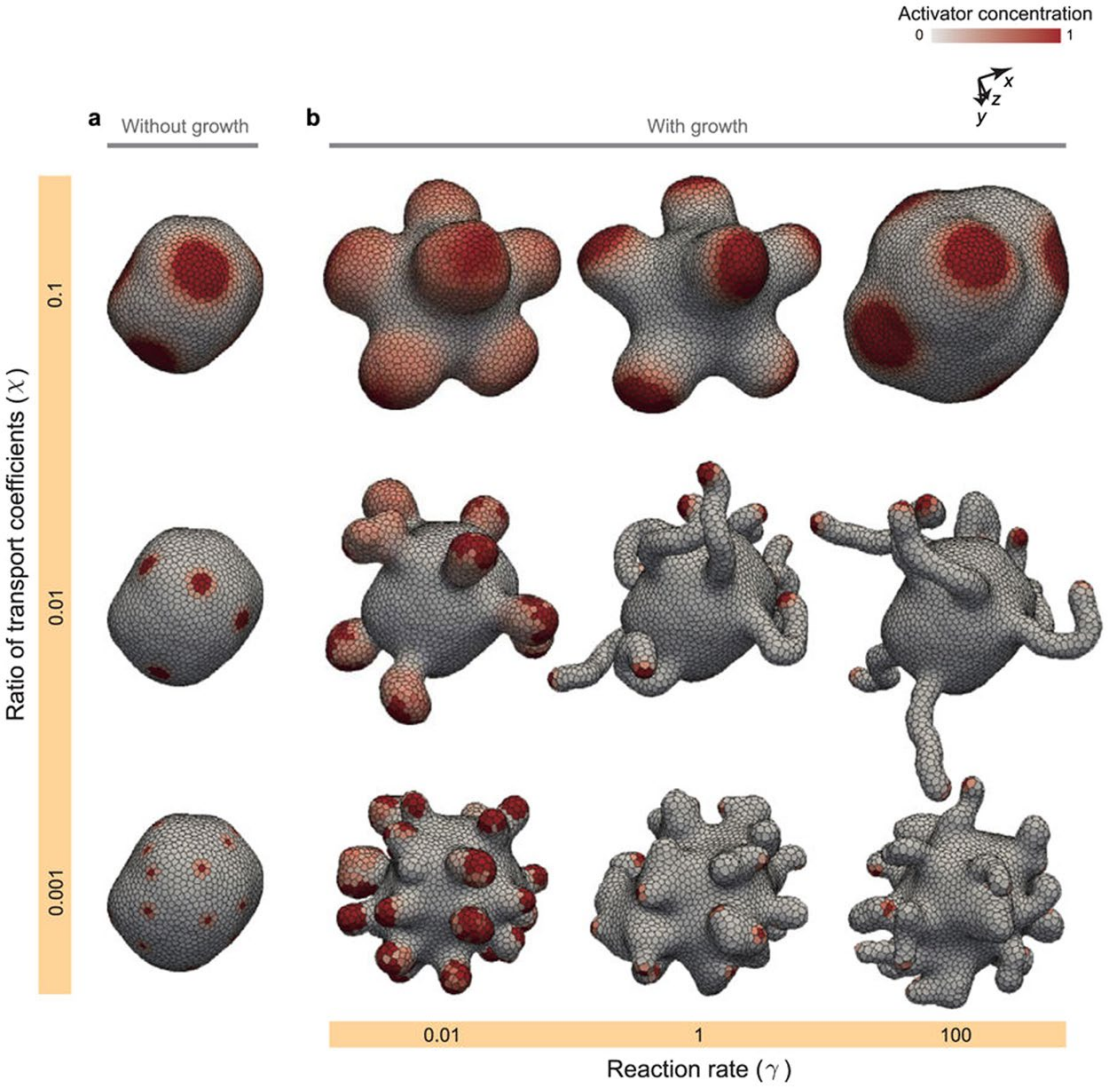
Morphology diagram. (**a**) Images of tissue patterns obtained by simulations without cell growth. (**b**) Morphology diagram of tissue shapes and patterns obtained by simulations with cell growth. In (**a** and **b**), cells are colored by their activator concentration. The steady tissue patterns in (**a)** were obtained by simulations during 2 cell cycles, in which physical parameters were set as *γ* = 100. The individual tissues in (**b**) were composed of about 4,000 cells, which were picked up on the growth process.

**Figure 2:**
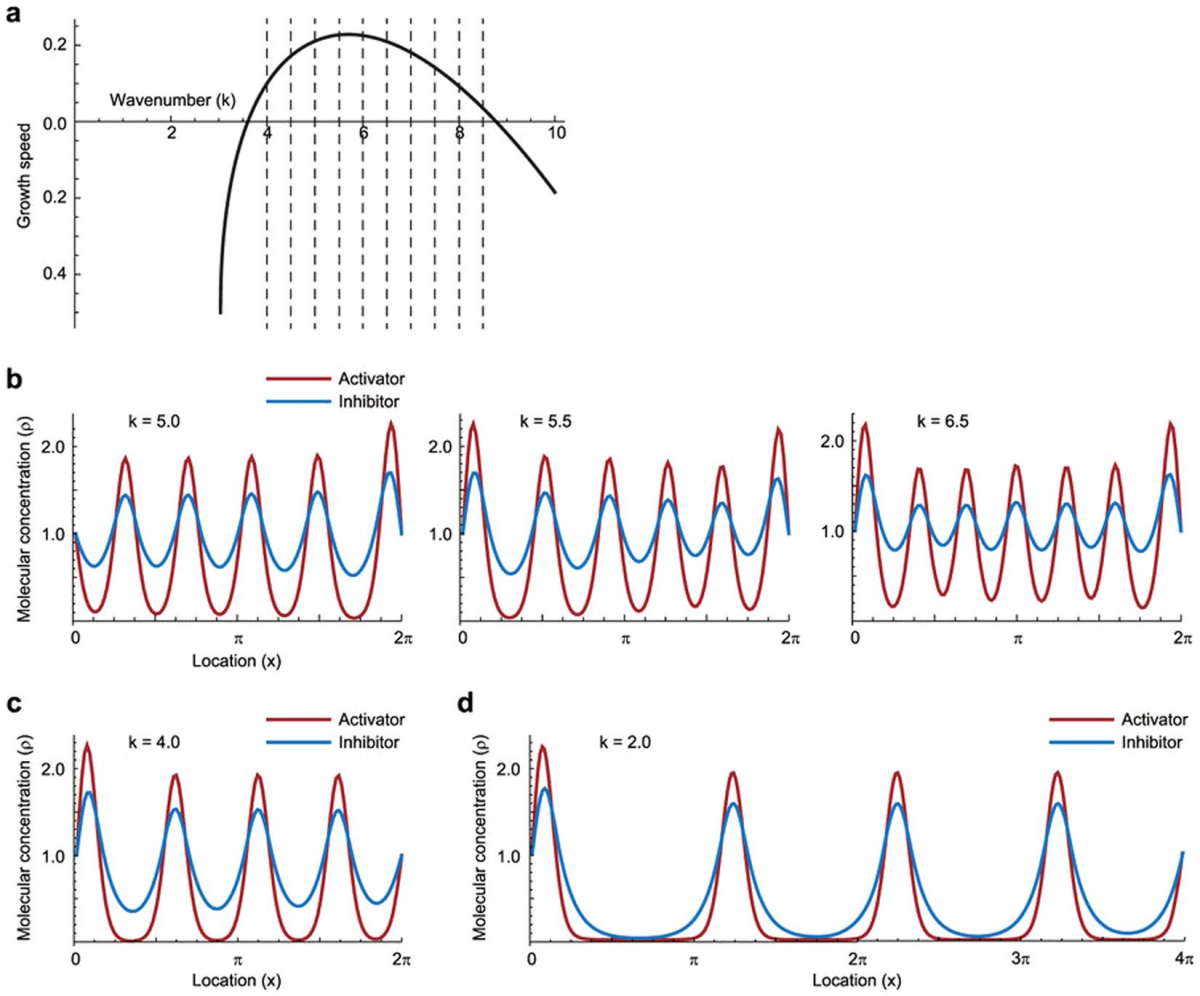
Multiple unstable mode of activator–inhibitor system in one-dimensional space. (**a**) Dispersion relation of the activator–inhibitor system. Growth speed of each wavenumber component is positive in the region of 4.0 ≤ *k* ≤ 8.5. Dashed lines indicate the possible wavenumber components. (**b**) Example results of the numerical simulations for the activator–inhibitor system with respect to the different initial conditions. The initial conditions were set as a random distribution with the uniform white noise, the boundary condition was set as ${{\rho }}_{{\rm{A}}}$ = ${\rho }_{{\rm{I}}}$ = 1 at *x* = 0, 2*π*, and physical parameters were set as ${D}_{{\rm{A}}}/\gamma$ = 0.01 and ${D}_{{\rm{I}}}/\gamma$ = 0.1. Red and blue lines indicate activator and inhibitor, respectively. (**c**) Result of the numerical simulation from the initial condition with dominant number of waves 4. (**d**) Result of the numerical simulation with domain growth from the initial condition with dominant number of waves 4. The domain size grew from 2*π* to 4*π*. In (**c** and **d**), the initial condition was set as periodic as ${{\rho }}_{{\rm{A}}}$ = ${\rho }_{{\rm{I}}}$ = 1 + sin4*x*.

Additionally, this Article contains typographical errors in the Modeling Example: Cell Growth Regulation by an Activator–inhibitor System and Results sections.

Under the subheading ‘Discrete Turing model for describing multicellular patterning’,

“Sect. 6 in Appendix”

should read:

“Appendix A”

Under the subheadings ‘Activator–inhibitor system’ and ‘Hysteresis in patterning emerges from the multiple unstable mode in activator–inhibitor system’,

“Sect. 7 in Appendix”

should read:

“Appendix B”

Under the subheading ‘Physical parameter setting’,

“Sect. 8 in Appendix”

should read:

“Appendix C”

Finally, under the subheading ‘Coupling patterning and deformation drives undulation, tubulation, and branching’,

“As described in Sect. 3.4, the size of activator regions should be approximately proportional to χ^1/4^ϕ^1/2^; hence, the size in the case of χ = 0.1 is expected to be about 1.8 times larger than that in the case of χ = 0.01.”

should read:

“Based on linear approximation, the size of activator regions should be approximately proportional to χ^1/4^ϕ^1/2^; hence, the size in the case of χ = 0.1 is expected to be about 1.8 times larger than that in the case of χ = 0.01.”

